# Evaluation of the safety and feasibility of electrochemotherapy with intravenous bleomycin as local treatment of bladder cancer in dogs

**DOI:** 10.1038/s41598-023-45433-4

**Published:** 2023-11-29

**Authors:** Marcelo Monte Mor Rangel, Laís Calazans Menescal Linhares, Krishna Duro de Oliveira, Daniela Ota Hisayasu Suzuki, Felipe Horacio Maglietti, Andrigo Barboza de Nardi

**Affiliations:** 1 VetCancer Veterinary Oncology, São Paulo, São Paulo Brazil; 2 School of Agricultural and Veterinary Sciences, São Paulo State University (UNESP) “Júlio de Mesquita Filho”, Jaboticabal, São Paulo Brazil; 3 VCLab Pathology, São Paulo, São Paulo Brazil; 4 Institute of Biomedical Engineering, Federal University of Santa Catarina (UFSC), Florianopolis, Santa Catarina Brazil; 5 Instituto Universitario de Ciencias de la Salud. Fundación Barceló-CONICET, Buenos Aires, Argentina

**Keywords:** Urological cancer, Cancer therapy, Translational research, Oncology

## Abstract

Local treatment of canine urothelial carcinoma (UC) of the bladder is a challenge. More than 90% of the cases invade the muscular layer, more than 50% develop on bladder sites with a difficult surgical approach and often requiring radical surgical procedures. This study aims to evaluate the safety and feasibility of electrochemotherapy (ECT) with intravenous bleomycin (BLM) as a local therapy for bladder UC. This prospective study included 21 dogs with spontaneous bladder UC. Regional/distant metastases and neoplastic infiltration of the serosa was considered the main exclusion criteria. We had no deaths during ECT or in the immediate postoperative period, and no suture dehiscence. Most dogs (19/21) developed mild adverse effects, whereas two dogs developed ureteral stenosis. Complete response (CR) was achieved in 62% of the cases (13/21), while partial response (PR) was achieved in 24% (5/21). The median survival and disease-free survival times were 284 and 270 days, respectively. Overall survival was significantly better in the dogs who achieved a CR. In conclusion, ECT was well-tolerated in dogs with UC, demonstrating its safety and feasibility. These data pave the way for new studies aimed at evaluating the effectiveness of ECT in canine bladder UC as a translational model for human disease.

## Introduction

Urothelial carcinoma (UC) is considered the most commonly diagnosed malignant bladder neoplasm, accounting for approximately 1.5–2% of all naturally occurring cancers, both in humans and dogs^1^. According to the World Health Organization staging system for canine bladder tumors, they are classified according to the degree of invasion into the bladder wall, being classified as T2 the tumors invading the muscle layer and T3 the tumors invading neighboring structures, as urethra and prostate (Supplementary Table 1)^2^. In humans, the staging system is similar, but muscle-invasive disease is classified as T3 or higher^2^. More than 90% of affected dogs present at T2 stage or higher (InvUC), which is most commonly located in the trigone of the bladder, extending down to the urethra in more than 50% of cases^3–5^. Therefore, the surgical approach for these tumors is a great challenge, as partial cystectomy is possible in only a few cases, mainly when the trigone is not involved^6–8^.

Total cystectomy is not usually performed in dogs because they are commonly in advanced stages of the disease and also because of the higher morbidity and mortality associated with the procedure^4,9–12^. It is important to highlight that even in the best case scenarios when partial cystectomy is possible, the tumor recurrence rate is still high, ranging from 66 to 100%^5,6,8,13,14^.

Radiation therapy is not commonly used for bladder cancer in dogs mainly because of its side effects, as reported in the literature^15–17^. However, newer radiation protocols have shown better tolerability, encouraging further investigations^18–20^.

Currently, medical therapy is the mainstay for InvUC in canines. The combination of chemotherapy with non-steroidal anti-inflammatory drugs (NSAIDs) improves clinical signs and provides disease control in 80% of the cases. However, it is not curative, and the response is temporary^7,21^. Recently, the identification of overexpressed specific proteins associated with UC progression in dogs had led to the investigation of targeted therapies, including BRAF, CCR4, HER-2 and EGFR inhibitors. Results from recent clinical trials of these drugs already yielded preliminary evidence of antitumor activity^22–24^.

In contrast, in humans, the superficial non-invasive form of bladder UC comprises approximately 75% of cases, with no predilection for specific anatomical sites of the bladder^1,25^. These features allow for a more feasible surgical approach, i.e. transurethral resection of the tumor. Despite this better scenario, high recurrence rates after surgery are also common, reaching up to 75% at five years, progressing to InvUC^25,26^. As in dogs, human InvUC also has potentially lethal behavior, requires aggressive treatment, and only 5% of patients with metastatic disease remain alive at 5 years^27,28^.

Therefore, preclinical animal studies have been conducted to investigate promising therapies for human bladder UC. Experimental mouse models have been used for many years in bladder cancer research. However, they lack the key features of the disease in humans and cannot accurately predict therapeutic outcomes^4,29,30^. Advances in translational research have proven that dogs with naturally occurring InvUC represent a highly relevant animal model for studying invasive disease in humans. These studies have identified notable similarities between dogs and humans with InvUC, including etiological factors, biological behavior, molecular features, and chemotherapeutic response rates^1,4,31^.

Considerable efforts are being made to develop less invasive and effective therapeutic approaches for the local treatment of human and canine bladder cancer as well as for the prevention of recurrence. Electrochemotherapy (ECT) is a local technique that combines the injection of antineoplastic drugs followed by the application of electrical pulses at the tumor site and margins, aiming to increase drug uptake by electropermeabilized cells and maximize its cytotoxic effect. This technique stands out for its high efficacy, low cost, and selectivity towards neoplastic tissues. The latter feature allows for the conservation of important anatomical structures, especially when tumors develop in sites with a difficult surgical approach^32–34^

The efficacy of ECT has already been proven in different types of cancers in humans and canines, including melanomas, carcinomas, mast cell tumors, and sarcomas^34–36^. Regarding its application in carcinomas, ECT has demonstrated significant efficacy in canine squamous cell, anal sac, and nasal carcinomas^36–39^ as well as in human squamous and basal cell carcinomas^40,41^. ECT has also been shown to be safe and effective for deep-seated tumors in humans, such as pancreatic and hepatocellular carcinomas^42,43^. Some studies have evaluated the efficacy of ECT in vitro and in vivo in cultured human bladder cancer cells and in rats with induced UC^44–47^. Recently, ECT treatment of dogs with naturally occurring bladder UC was first reported in three dogs by Rangel et al.^48^ with promising results.

This study aimed to evaluate the safety and feasibility of ECT as a local treatment for canine bladder UC. We also evaluated the local antitumor effect of the therapy, paving the road for future clinical trials in human medicine.

## Results

### Case data

Between 2016 and 2021, 26 dogs were initially enrolled for the study, however, a total of 21 dogs met the criteria for definitive inclusion. The demographics of the treated dogs, and the data regarding tumor features and treatment outcomes for each case are described in Tables 1 and 2, respectively.

**Table 1:** Demographic characteristics of the dogs treated.

Characteristics of 21 dogs	Mean (range) (%)
Age (years)	11.4 (8–16)
Sex ratio (F:M)	13:8
Body weight (kg)	8.69 (2.9–27)
Breeds	
Shih-tzu	4 (19%)
Poodle	4 (19%)
Lhasa Apso	3 (14.2%)
Maltese	2 (9.5%)
Dachshund	2 (9.5%)
Pug	1 (4.7%)
Schnauzer	1 (4.7%)
Yorkshire	1 (4.7%)
Labrador retriever	1 (4.7%)
French bulldog	1 (4.7%)
Crossbreed	1 (4.7%)

**Table 2:** General case information, tumor features and treatment outcome.

Case no.	Tumor location**	TNM	No. of ECT sessions	Final local response		Local relapse	VCOG-CTCAE grade	DFS*	ST*	Cause of death
1	Cranial-dorsal	T_1_N_0_M_0_	1	PR		–	1	–	517	Local progression (UC)
2	Trigone	T_2_N_0_M_0_	3	CR		Yes	1	790	866	Other tumor
3	Apex	T_2_N_0_M_0_	1	CR		No	1	30	30	Perforated gastric ulcer
4	Cranial-dorsal	T_2_N_0_M_0_	1	CR		No	1	147	147	Peliosis hepatis
5	Trigone	T_2_N_0_M_0_	3	CR		No	1	981	1021	CKD
6	Apex	T_2_N_0_M_0_	1	CR		Yes	1	353	740	Mammary tumor
7	Trigone	T_2_N_0_M_0_	2	CR		No	1	199	199	Pelvic sarcoma
8	Trigone	T_2_N_0_M_0_	1	CR		No	1	166	166	Pulmonary metastasis (UC)
9	Trigone	T_2_N_0_M_0_	2	CR		Yes	1	257	595	CKD
10	Trigone	T_2_N_0_M_0_	1	CR		No	1	978	978	Mast cell tumor
11	Trigone	T_2_N_0_M_0_	2	CR		Yes	3	244	1015	CKD
12	Trigone	T_2_N_0_M_0_	1	UR		–	1	–	20	AKI
13	Trigone	T_2_N_0_M_0_	1	UR		–	1	–	7	Undetermined
14	Trigone	T_2_N_0_M_0_	2	PR		–	1	–	283	Ehrlichiosis
15	Trigone	T_1_N_0_M_0_	1	CR		No	1	495	515	Cardiomiopathy
16	Trigone	T_2_N_0_M_0_	2	PR		–	1	–	284	Sepsis
17	Trigone	T_2_N_0_M_0_	2	PR		–	3	–	168	Sepsis
18	Trigone	T_2_N_0_M_0_	1	PR		–	1	–	155	Local progression (UC)
19	Trigone	T_2_N_0_M_0_	1	CR		No	1	499	499	Alive
20	Cranial-dorsal	T_2_N_0_M_0_	3	CR		Yes	1	130	571	Alive
21	Trigone	T_2_N_0_M_0_	1	UR		–	1	–	34	Ehrlichiosis

*ECT* electrochemotherapy, *VCOG*-*CTCAE* veterinary cooperative oncology group—Common terminology criteria for adverse events, *PR* partial response, *CR* complete response, *UR* unassessed response, *ST* survival time, *DFS* disease-free survival, *UC* urothelial carcinoma, *CKD* chronic kidney disease, *AKI* acute kidney injury.

*Days, **Inside the bladder.

Complete blood count and biochemical profile performed prior to ECT, including BUN and creatinine, were in normal range for all the included dogs.

Twelve of the 21 dogs (57%) underwent one ECT session, six (29%) underwent two sessions, and three (14%) underwent three sessions. Overall, the interval between sessions ranged from 33 to 70 days and was individualized for each case, depending on the response to the treatment. The mean interval between the first and second session was 52 days, and between the second and third session was 45 days.

### Intraoperative histopathological evaluation

**Figure 1:**
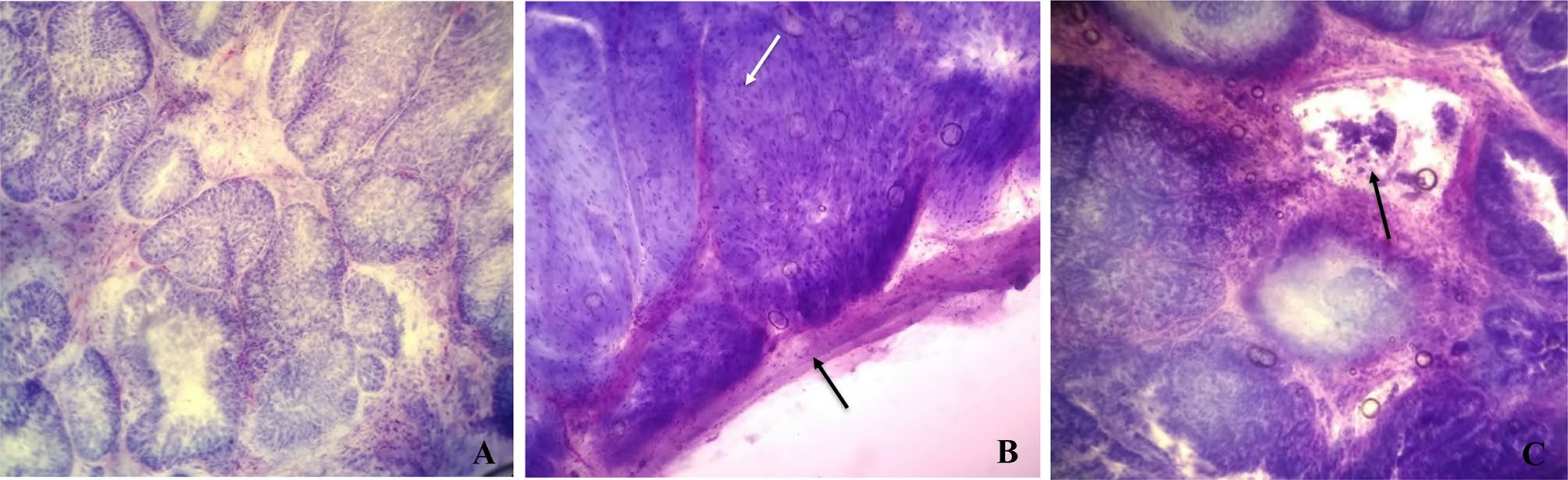
Intraoperative histological photomicrograph by frozen section of bladder UC (case #15) (**A**) Central region of a papillary UC (toluidine blue). (**B**) Muscular (white arrow) and serosal (black arrow) layers of the bladder free of neoplastic infiltration (toluidine blue). (**C**) Lymphatic infiltration of tumoral cells (black arrow) (toluidine blue).

**Figure 2:**
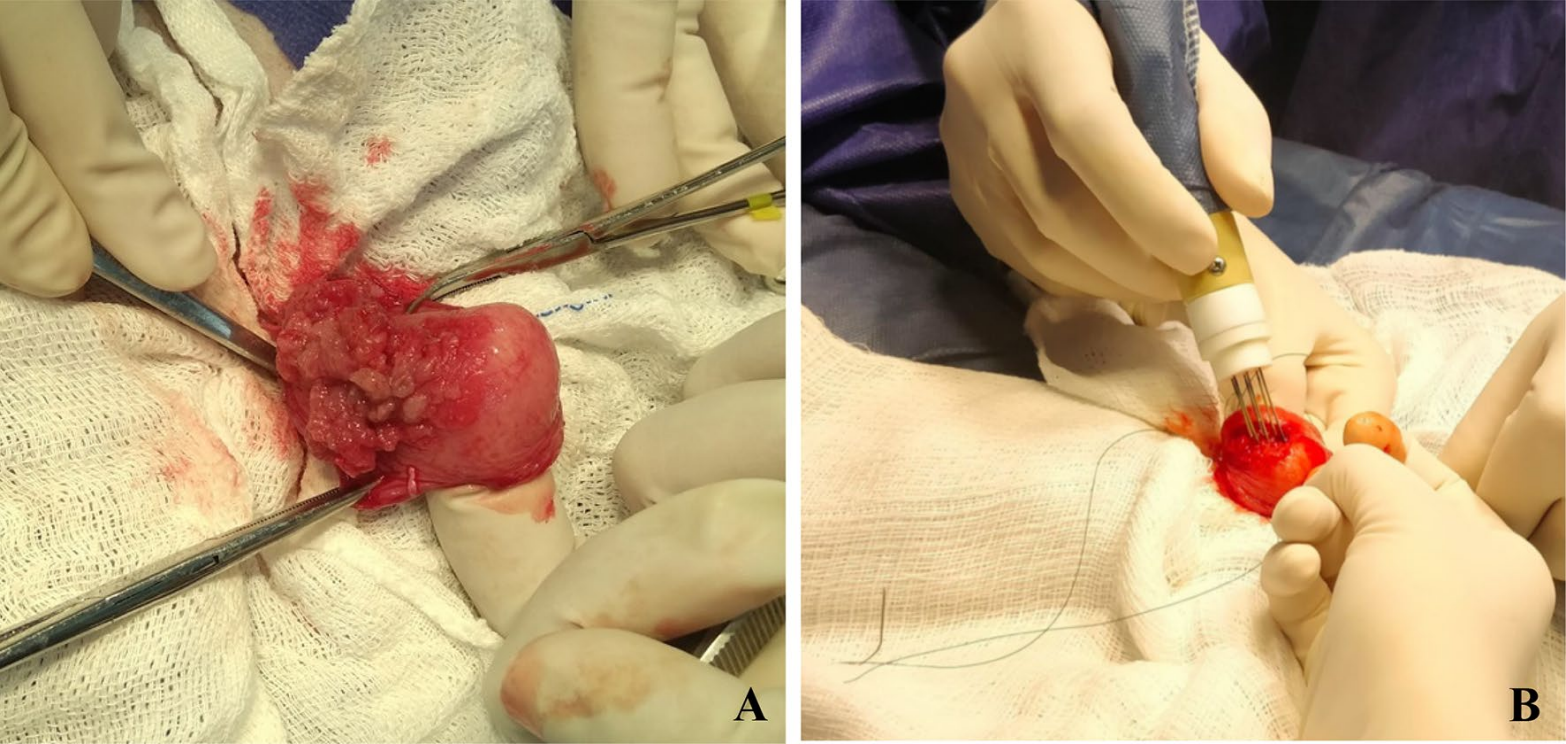
Case #5 (**A**) Tumor exposure with eversion of vesical mucosa following cistotomy. (**B**) ECT application in the bladder.

Intraoperative histopathological analysis was performed for the 26 dogs initially enrolled (Fig. 1A). Neoplastic infiltration of the serosa was not identified in 21 cases, which received ECT treatment immediately after evaluation (Fig. 1B and 2; Supplementary Video 1). Conversely, five dogs presented serosal involvement, which were excluded from the study and redirected to other therapies.

Information about other histopathological features are described in Table 3. High grade papillary UC represented 76% of the cases (16/21) and the majority of them were classified as grade 3 (13/16). Three cases had non-papillary tumors and all of them were high grade. Only one case was classified as UC with divergent differentiation (microcystic). Inflammation was present in almost all cases (95%; 20/21) and lymphatic invasion was identified in four cases (19%) (Fig. 1C).

**Table 3:** Histopathological features of all the cases evaluated in this study.

Histopathological features	% (n)
Growth pattern	
Papillary UC	81% (n = 17)
Non-papillary UC	14% (n = 3)
Tumor differentiation	
Conventional UC	95% (n = 20)
Divergent UC (microcystic)	5% (n = 1)
Grade*	
Low-grade (grade 1)	10% (n = 2)
Low-grade (grade 2)	14% (n = 3)
High-grade (grade 3)	62% (n = 13)
High-grade (grade 4)	14% (n = 3)
Inflammation	
Present	95% (n = 20)
Absent	5% (n = 1)
Lymphatic invasion	
Present	19% (n = 4)
Absent	81% (n = 17)

*According to Cheng et al.^69^ and Meuten^70^.

### Safety evaluation

None of the dogs died during the surgical procedure, ECT treatment, or in the immediate postoperative period. Furthermore, none of the dogs had suture dehiscence in the bladder or uroperitoneum. All the dogs were evaluated for adverse events (Table 4). Nineteen dogs (90%) presented with mild adverse effects during the postoperative period, including pollakiuria, stranguria, dysuria, and transitory urinary incontinence. The degree of toxicity for these dogs was classified as grade 1 according to the “Veterinary Cooperative Oncology Group-Common Terminology Criteria for Adverse Events” (VCOG-CTCAE v2)^49^.

**Table 4:** Adverse events and treatment related toxicity (veterinary comparative oncology group criteria) in dogs in the clinical trial.

Adverse event	Number of dogs (%) VCOG grade
Pollakiuria, dysuria, stranguria	21 (100%) 1
Urinary incontinence	21 (100%) 1
Ureteral stenosis	2 (9.5%) 3

**Figure 3:**
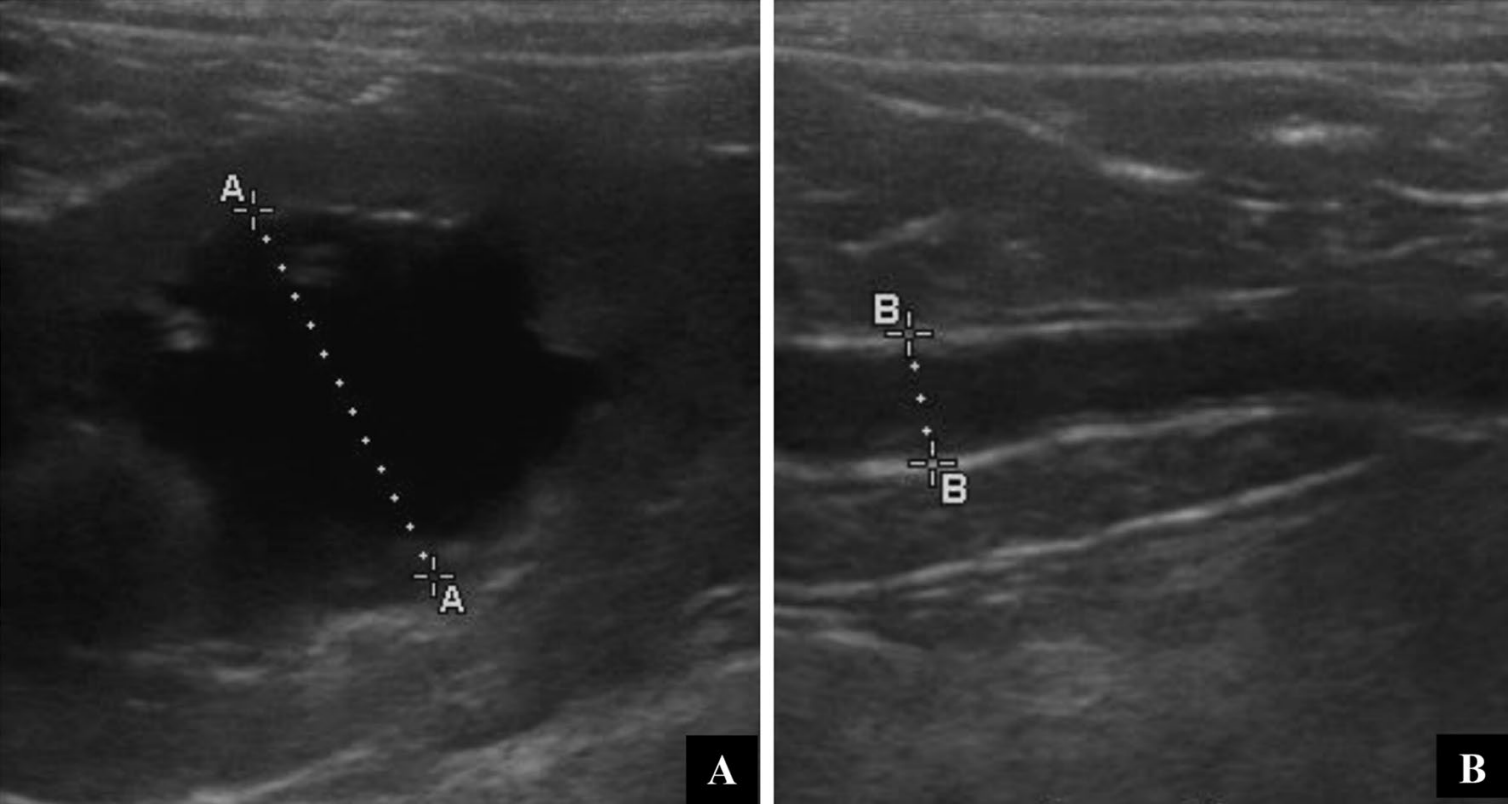
Ultrasonographic evaluation of the urinary tract of case #17. (**A**) Moderate to severe pyelectasis of right kidney; renal pelvis measuring 2 cm. (**B**) Ureter dilation (i.e. megaureter) due to stenosis of the ureteral ostium, after 56 days from the second ECT session.

Two dogs had more serious complications (VCOG = 3), developing ureteral stenosis 56 and 69 days after the procedure. These dogs presented ultrasonographic alterations consistent with ureter dilation, i.e. megaureter, pyelectasis, and/or hydronephrosis (Fig. 3). Thereafter, they underwent a surgical procedure for the placement of a temporary “double-J” ureteral stent which was removed after recovery of ureteral patency.

Life-threatening abnormalities on blood tests were detected during follow-up in four dogs, who died within 34 days (cases #3, #12, #13, #21) due to causes unrelated to the ECT. These alterations included anemia, thrombocytopenia, leukocytosis, and elevated levels of serum creatinine and BUN, and were associated with their respective causes of death (Table 2).

### Quality of life

According to the quality of life questionnaire filled by the owners and the oncologist’s clinical evaluations, all 19 dogs who developed mild adverse events had no relevant negative impact on their quality of life.

As expected, the two dogs that developed ureteral stenosis manifested a more severe alteration of their life quality, including a higher level of pain and changes in behavior. However, this negative impact on the quality of life was transitory, lasting until surgical resolution of the complication. The procedures were well tolerated by the dogs, who completely recovered their baseline quality of life score according to subsequent assessments.

### Cause of death

Three dogs died of disease progression. Of these, two dogs developed local progression of the tumor inside the bladder (case #1) and to the prostate (case #18), and the other dog developed distant metastatic spread to the lungs (case #8).

Sixteen dogs died of causes unrelated to the disease or the ECT, which included mainly other types of neoplasia (4/16; 25%), and chronic kidney disease (3/16; 18.7%). The remaining causes of death included ehrlichiosis, sepsis, pulmonary thromboembolism, peliosis hepatis, perforated gastric ulcer, acute kidney injury and cardiomyopathy (Table 2). It was not possible to determine the cause of death in one dog.

Necropsy was performed only in two dogs (case #3 and #4). In the remaining cases it was not performed due to owners’ refusal, and the cause of death was presumed according to the clinical condition and blood and imaging tests.

### Local response

**Figure 4:**
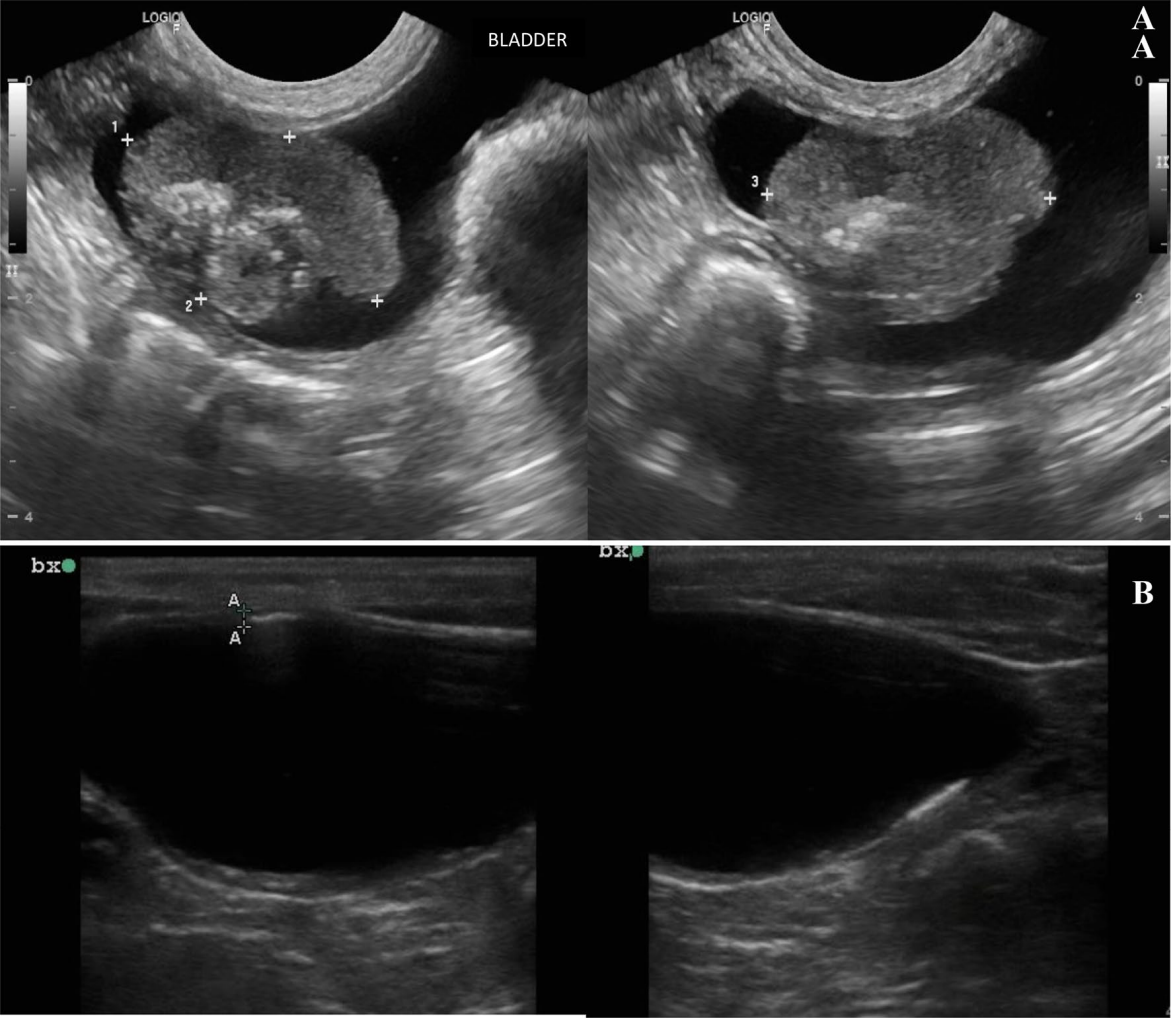
Local response assessment by ultrasonography of case #15. (**A**) Bladder tumor before the first ECT session. (**B**) Complete tumor remission after one ECT session.

**Figure 5:**
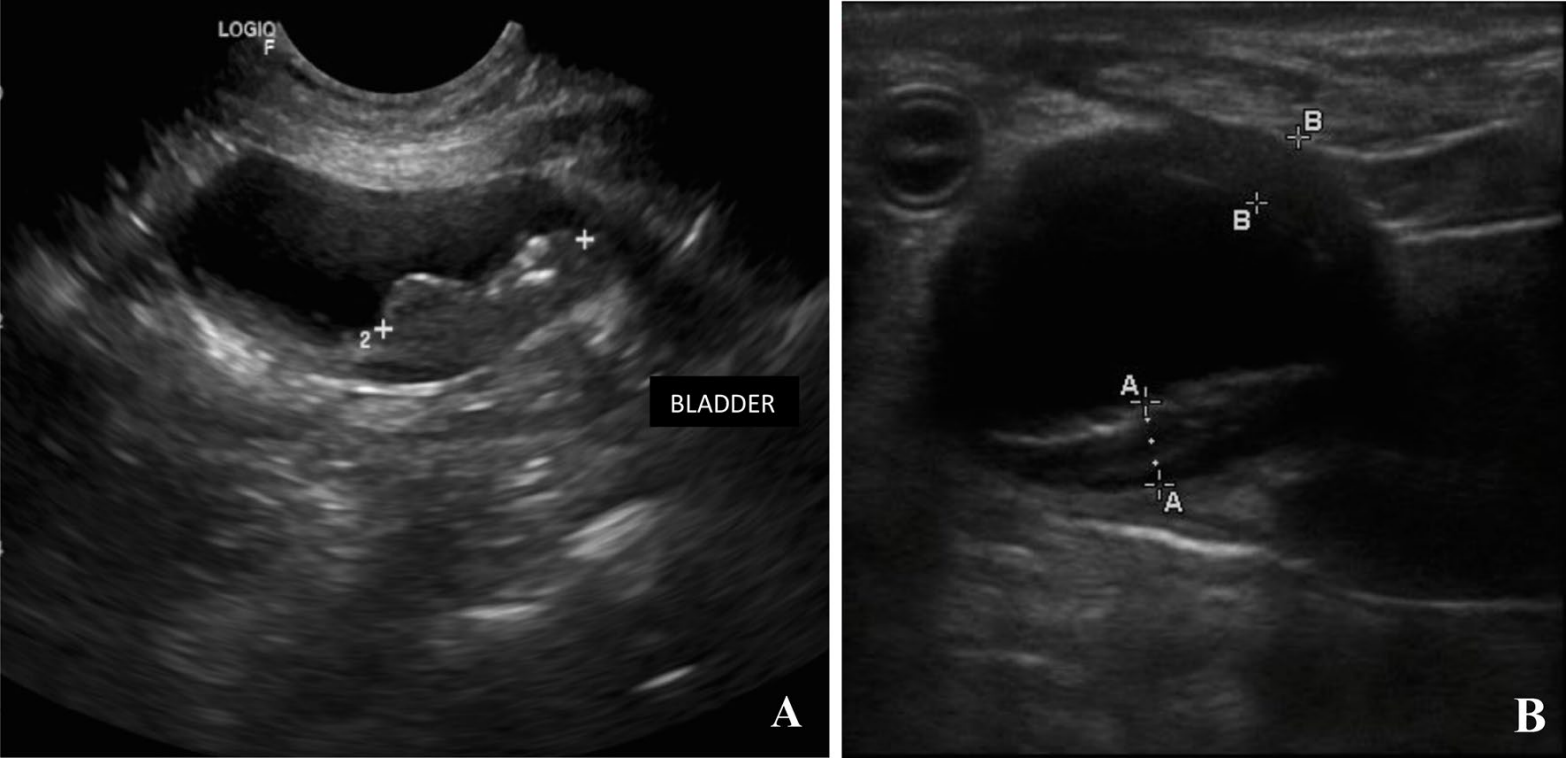
Local response assessment by ultrasonography of case #17. (**A**) Bladder tumor before the first ECT session. (**B**) Partial tumor remission after two ECT sessions.

Sixty-two percent of the dogs (13/21) achieved a complete response (CR) (Fig. 4), and 24% (5/21) had partial response (PR) (Fig. 5). An objective response rate (ORR) of 86% was achieved. The remaining three dogs were not evaluated, as the minimum time required to apply the RECIST criteria was not met.

The local response was not associated with sex (Fisher’s exact test, p = 0.4) or tumor location (Fisher’s exact test, p = 0.4).

Among the dogs who achieved a CR, most of them achieved it in one session (77%). Among the dogs who required two sessions (15%), a median of 56 days (range 22–71) passed between the two sessions. For the dogs who required three sessions (8%), a median of 43 days (range 33–60) passed after the second session.

Microscopic neoplastic foci were identified by additional surgery in five dogs who had a CR, which underwent a new ECT session. However, the owners of the remaining eight dogs with CR refused any additional surgery.

Of the five dogs who achieved PR, no additional ECT sessions were performed because of the owner’s refusal. Two dogs showed local disease progression.

The median duration of response (DOR) was 270 days (average 371, range 30–981 days). Tumor recurrence occurred in five dogs (38%) in which CR was achieved, and the median time to its development was 257 days (average 354, range 170–790 days) (Table 2).

At the end of the study, two dogs remained alive, one with CR and the other with tumor recurrence. Among the 19 dogs who died, six (31.5%) died without tumor evidence (CR), 10 (52.6%) died with macroscopic or microscopic tumor evidence, and three (15.7%) died before the period of response evaluation (Table 2).

### Survival data

**Figure 6:**
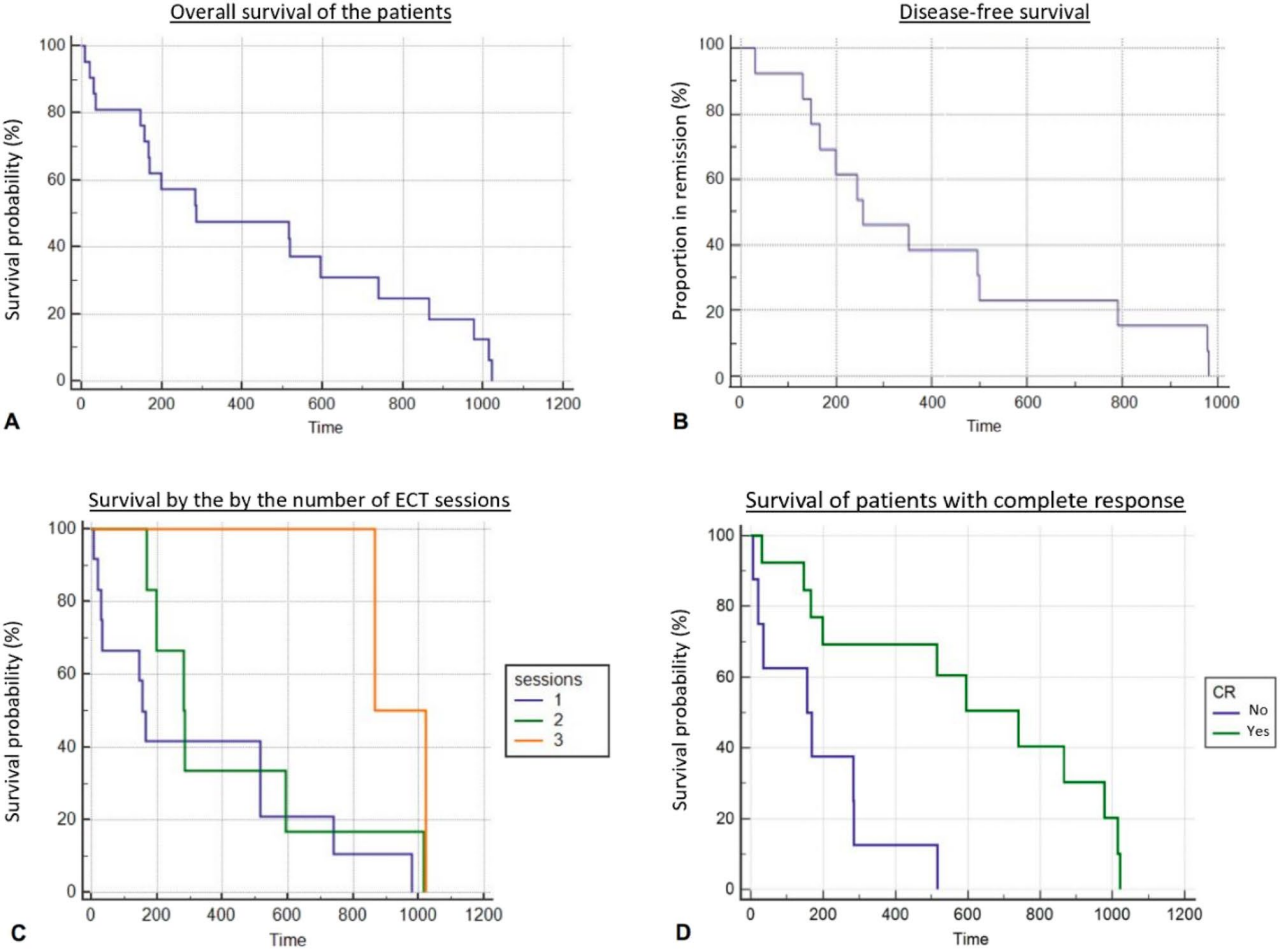
Kaplan–Meier curves. (**A**) Overall survival time (n = 21). (**B**) Disease-free survival (n = 13). (**C**) Survival time by the number of ECT sessions performed (Log-rank test, p = 0.0665). (**D**) Survival time when a complete response was achieved (Log-rank test, p = 0.0035).

Considering the dogs who achieved a CR (n = 13), the median disease-free survival (DFS) was 270 days (average 405, range 30–981 days) (Fig. 6B). DFS was not associated with sex (p = 0.2839), tumor location (p = 0.2552), or number of sessions (p = 0.1711). The median progression-free survival time (PFS) was 283 days (average 281, range 155–517 days), for the dogs who achieved PR (n = 5).

The median overall survival (OS) of the dogs was 284 days (average 420, range 7–1021 days) (Fig. 6A). Sex (p = 0.0355), number of sessions (p = 0.0622; Fig. 6C), and tumor location (p = 0.5590) were not associated with OS. However, obtaining a CR was associated with longer OS (Log-rank test, p = 0.0035; Fig. 6D), as expected.

## Discussion

To date, two preliminary studies have reported the application of ECT in canine bladder UC; however, both studies used a small series of cases^48,50^. Considering the anatomical characteristics of the urinary bladder as well as the possible side effects associated with ECT, the present study evaluated the safety and feasibility of this technique for the treatment of bladder UC in 21 dogs. Additionally, the local ECT antitumor effect was preliminarily assessed.

Demographic data regarding sex predisposition, age at diagnosis, and reproductive *status* are in agreement with previous studies^1,7,21^. Shih Tzu and Poodle (38%) were the most affected breeds in this study, with no occurrence of the most known predisposed breeds. This fact may be related to differences in racial distribution between the canine population in Brazil, the United States, and Europe.

Intravenous bleomycin (BLM) was selected as the antineoplastic drug for ECT in this study. BLM possesses a selective mechanism of cell death, and its systemic administration allows for a more homogenous distribution through tissues. In addition, electropermeabilization promotes BLM intracellular uptake by a factor of 700-fold^32,33,51–53^. Once inside the nucleus, BLM leads to single- and double-strand breaks in the DNA. This damage accumulates as the cells proliferate, and after a threshold is reached, cell death occurs by mitotic cell death process. This mechanism confers BLM with unique selectivity towards rapidly dividing cancer cells. In turn, cells from healthy tissues (such as those from tumor margins) are quiescent and slow or non-dividing, and remain viable even if electropermeabilized^32,52^.

Thus, the process of cell death caused by ECT occurs in any tissue where cancer cells infiltrate. Consequently, ECT application in bladder tumors that infiltrate the serosa would entail significant risks of bladder rupture and uroperitoneum, as the serosa is the last layer of the bladder lining. Therefore, the non-involvement of serosa was a crucial inclusion criterion in this study.

In addition to the evaluation of tumor invasion, other relevant histopathological information was obtained from each case. Papillary infiltrative UC was considered the most prevalent histological subtype in this study, agreeing with the results of Govoni et al.^54^. Regarding tumor differentiation, 20 cases (95%) in this study were classified as conventional UC, while only one case was classified as divergent UC. Similarly, in the study of De Brot et al.^1^, 90% of the dogs had conventional UC, and 6% divergent UC.

Recently, lymphatic invasion has been proposed as a relevant negative prognostic factor for canine UC^54^. In the present study, only four cases showed lymphatic invasion and due to this small sample size, it is difficult to determine whether this feature affected the OS of the dogs.

None of the cases in this study died during the procedure or in the immediate postoperative period, and there were no occurrences of bladder rupture, uroperitoneum, or suture dehiscence. Similarly, Rangel et al.^48^ performed ECT with intravenous BLM in two dogs with bladder UC and adopted the non-involvement of the serosa as a safety criterion. None of their dogs developed any of these complications.

The vast majority of dogs in this study developed mild adverse effects (90%; VCOG = 1), which had already manifested at the first presentation. However, transient urinary incontinence (lasting 7–86 days) was the only adverse effect not previously reported. Likewise, Rangel et al.^48^ also reported transient urinary incontinence in dogs after the treatment, lasting 56 and 76 days. The development of this condition may be due to transient inflammation and edema in the bladder mucosa, which are expected to be caused by ECT, leading to nerve stunning and involuntary muscle spasms.

Two dogs (9.5%) developed ureteral stenosis at 56 and 69 days after the second ECT session (VCOG = 3). They were treated surgically by placement of a “double-J” stent with good tolerability. Ureteral stenosis might be attributed to scarring fibrosis resulting from tumor death, which is an expected effect after ECT. However, as the amount of fibrotic tissue increases, the risk of stenosis increases. In front of this, we believe that the tumor proximity to the ureter could be a risk factor for the development of ureteral stenosis. Additional care should be taken in those patients as well as more frequent clinical and imaging reassessments.

One of these dogs (case #17) achieved a PR and developed ureteral stenosis after 56 days from the second session of ECT. At the time of the stent placement, the tumor was not macroscopically identifiable, however, there was a thick area of fibrosis in the bladder wall, close to the ureteral ostium, leading to its stenosis. Intraoperative pathology evaluation of this area revealed the presence of superficial microscopic UC confined to the mucosa associated with inflammation. However, we did not perform a third session of ECT because of the concurrent scarring process in the same area, minimizing the amount of inflammatory tissue (Supplementary Fig. 1).

Studies evaluating partial or total cystectomy in dogs with bladder UC have found more significant complication rates than we did (range 43–70%), including suture dehiscence, ureteral obstruction, pyelonephritis, and uroperitoneum^6,11,14^.

In particular, partial cystectomy, with or without adjuvant chemotherapy, have reported death rates due to disease progression ranging from 45 to 91%^6,8,14^. In the present study, only three dogs (14%) died of UC, one due to distant metastasis and the other two due to local tumor progression. The dog who died due to tumor progression inside the bladder (case #1) achieved a PR and died 517 days after the only ECT session performed. No additional sessions or other treatments were performed due to owner refusal. However, the survival time was above the average for dogs treated with conventional therapies (surgery and/or chemotherapy)^6,8,55,56^.

The other case who died due to local progression (case #18) was an intact dog who presented prostate enlargement with mineralization foci, non responsive to castration. Prostate cytological examination was inconclusive and the owner declined to perform prostate biopsy and necropsy after death. Abdominal ultrasound did not reveal urethral abnormalities. Although it is difficult to determine if it was a primary prostate tumor or a local spread of the bladder tumor to the prostate, we considered it as a local progression to the prostate, as the primary tumor was a grade 4, infiltrative UC with lymphatic invasion. In this study only one dog (case #8) developed distant metastasis to the lungs, identified by chest x-rays, meaning a metastasis rate of 4.7% In contrast, according to a study of Knapp et al.^5^, the rate of distant metastasis at the time of death was 49% among 80 dogs with bladder UC. This discrepancy could be attributed to the earlier stages of the disease in the present study. Another hypothesis may be attributed to the potential efficacy of ECT in eliminating cancer cells at the primary tumor site, decreasing the probability of metastasis. However, future studies including a control group could confirm this.

The remaining 16 cases died from causes unrelated to UC. The dogs included in this study were old with multiple comorbidities. The treatment of bladder cancer provided very good results and prevented the death of the animals from the disease. However, other concomitant pathologies, i.e. cardiac alterations, gastric ulcers, other tumors, among others, continued progressing and ended up provoking their death.

The case with undetermined cause of death (case #13) had a history of hyperadrenocorticism and diabetes, controlled with trilostane and insulin applications at home. No complications occurred during the ECT procedure and during the first 24 h of hospitalization. However, the dog was withdrew from the clinic by the owner’s decision before the completion of the minimum 48 h stay. After a few days the dog presented signs of dyspnea leading to a sudden death. The owners declined necropsy, so it is impossible to determine accurately the cause of death. We consider that the death is not related to the ECT procedure because abdominal ultrasound was unremarkable for urinary tract abnormalities and the dog was urinating normally before death. The cause could be attributed to pulmonary thromboembolism, as a complication of hyperadrenocorticism, considering the sudden clinical signs. Dogs with hyperadrenocorticism have an increased risk of developing blood hypercoagulability due to the increased procoagulant activity and/or decreased fibrinolysis, which can culminate in pulmonary thromboembolism^57,58^.

The case which died due to acute kidney injury (AKI; case #12) had a previous history of chronic kidney disease, however, the renal panel (BUN and creatinine) and urinalysis, performed prior to ECT, were unremarkable, as well as the dog was in good general condition. We believe that this dog may have developed an acute injury to the kidneys due to an aggravation of the previous CKD related to the use of NSAIDs during the postoperative period. Although AKI is not reported as a complication of ECT, caution should be taken with elderly dogs with some grade of kidney disease.

Lymph node involvement was assessed through fine-needle aspiration and intraoperative cytology. Even apparently normal nodes were evaluated. Although histopathology is the gold standard for definitive diagnosis of lymph node metastasis, we elected cytological evaluation because it is faster, either for sample collection or intraoperative evaluation, and it is associated with lower intraoperative complication rates. It is important to note that all dogs who had regional lymph node metastasis were also staged as T2 with serosa involvement or T3 during intraoperative evaluation, and consequently excluded from the study.

Local spread of tumor cells is also a concern in dogs with bladder UC who undergo cystotomy. Higuchi et al.^59^ and Marvel et al.^6^, reported the occurrence of abdominal wall metastasis during a follow-up at 121 and 230 days respectively. We had longer follow-up times of around 1000 days, and in contrast, none of the dogs in the present study developed this complication after cystotomy and ECT. We attribute this to the careful manipulation of the bladder and to the fact that the whole bladder was treated with ECT.

Regarding local response, out of the 21 dogs treated in this study, 13 (62%) achieved CR, 5 (24%) had PR, and 3 (14.3%) died of unrelated causes before the minimum period for response. This overall response rate (86%) was greater than those reported in studies evaluating different chemotherapy protocols for dogs with bladder UC, ranging from 3 to 71%, in which partial responses were predominant^7^. However, to confirm this, comparative studies are needed.

For the assessment of local response, ultrasound images sections obtained from each examination should be standardized, for ideal comparative purposes. However, as a limitation of the study, we could not standardize the cross-sections of the images depicted in the ultrasound report for some cases, as we can see in Fig. 4A (transverse section) and 4B (sagittal section).

The median DOR obtained was 270 days (average 371, range 30–981 days), which was higher than the 235 and 85 days obtained by Marvel et al. and Bradburry et al.^6,8^, respectively. The median DOR of this study was also higher than that reported in several studies evaluating medical therapy, which ranged from 96 to 199 days^60–62^.

One of the greatest challenges in treating bladder UC is the high recurrence rate after partial cystectomy, which ranges from 66 to 100% even when the tumor is resected with complete histological margins^6,8,14^. This finding may be explained by the phenomenon called “field cancerization effect,” which hypothesizes that bladder urothelium is uniformly exposed to carcinogens through the urine, making it more susceptible to the development of multiple microscopic foci of malignant transformation, ultimately leading to macroscopic UC^6,8,63^.

The recurrence rate of this study is 38%, which is considerably inferior when compared to the aforementioned studies. The median time to recurrence in these dogs was 257 (average 354, range 130–790 days) which is longer than the one reported by Marvel et al. and Bradburry et al.^6,8^ (101 and 85 days, respectively). It is important to note that in these two studies, medical therapy (chemotherapy and/or NSAIDs) was initiated after surgery. However, in the present study, ECT was performed as the sole treatment for all 21 dogs, indicating efficient disease control even without medical therapy. In the study of Bradburry et al.^8^, dogs predominantly in stage T2 were included as were in this study. In the study of Marvel et al. the TNM stage of the dogs was not reported.

One of the factors that may have contributed to the lower recurrence rate in this study might be attributed to the treatment of the entire bladder inner wall with ECT using intravenous BLM. This approach could lead to the destruction of microscopic neoplastic foci along the urothelium, minimizing the development of tumor recurrence and the risk of tumor implantation in other regions of the bladder. This is possible thanks to the selective mechanism of action of BLM, which leads to the preservation of the healthy urothelium^32,33,52^.

Regarding survival data, studies evaluating partial cystectomy in bladder tumors away from the trigone have reported a median OS varying from 348 to 498 days^8,13,14^. For most dogs in these studies, adjuvant chemotherapy and/or NSAIDs were combined with surgery, making it difficult to determine whether this association resulted in an increase in OS. In contrast, the median OS achieved by the dogs in this study was 284 days (average 420, range 7–1021 days), which is lower to those obtained in the aforementioned studies^8,13,14^. However, it is important to consider two main points in the present study. First, ECT was not combined with any other adjuvant therapy. Second, the majority of cases (76%) had tumors located in the bladder trigone. Tumors in this location naturally have a worse prognosis and shorter OS because of the complex surgical approach. Furthermore, tumor location was not correlated with DFS and survival time in the present study.

The median OS achieved was similar or superior to that obtained in studies that evaluated the use of chemotherapy (and/or NSAIDs) as a single treatment, with median OS ranging from 108 to 338 days^55,56,60,62,64,65^. In regard to targeted therapies, clinical trials with single-agent vemurafenib (BRAF inhibitor), mogamulizumab (CCR4 inhibitor) and lapatinib (HER-2 and EGFR inhibitor), alone or in combination with piroxicam, resulted in median OS of 354, 474 and 435 days, respectively^22–24^. These survival outcomes were very promising, encouraging further research to compare the effectiveness of targeted therapies with ECT, alone or in combination. In addition, there is an evidence of higher PDGFR-ß expression in bladder UC samples comparing with normal bladder and cystitis samples^66^, suggesting a potential clinical use of toceranib phosphate (VEGFR-2 and PDGFR-ß inhibitor). In a retrospective study^67^, the use of single-agent toceranib phosphate in dogs with UC resulted in a clinical benefit of 86.7%, however, the median OS was 149 days.

The time between ECT sessions varied in each case depending on the individual response, and usually ranged between 30 and 60 days. During the follow-up visits, it was noted that dogs who underwent a new procedure at shorter time intervals (33–40 days) had a greater amount of necrotic tissue in the tumor region, which remained from the previous session. This fact can be explained by the mitotic cell death caused by BLM, which is a slower process that relies on the replication rate of the tumor cells. This varies among tumors taking a longer time to achieve a response in tumors that replicate slower^51–53^. The current recommendation suggests that new ECT sessions should be performed when macroscopic evidence of the tumor (or relapse) is seen^33^. In this study, however, as we had intraoperative histopathological analysis, we indicated additional procedures even when macroscopic CR is achieved. In summary, according to our experience and considering the time to tumor response as an individual and variable factor, it is possible to determine the preferable interval between ECT sessions to range from 45 to 60 days.

In conclusion, ECT is a safe and feasible therapy for dogs with T1 and T2 bladder UC without serosal, urethral, or ureteral involvement or metastasis. The therapy was well tolerated by the dogs, providing significant local control of the disease along with a good quality of life. Regarding its antitumor efficiency, the results obtained in this study are promising; however, they are still preliminary. Further prospective studies should be conducted to evaluate the efficiency of ECT in bladder tumors, using a larger sample size of dogs and performing comparative analysis between different treatment groups (ECT vs. conventional therapy).

### Limitations

Although the primary aim of this study was to evaluate the safety and viability of ECT in canine bladder UC, one of its limitations is the lack of a control group comparing ECT with conventional treatments for bladder UC. The absence of a control group is due the fact that all dogs presented with bladder UC, that met the inclusion criteria, were treated with ECT. Another limitation of this study is the small number of dogs requiring two, and three sessions of ECT. For this reason, the comparison of the survival times among these groups is difficult. A case series with more dogs is needed to address this aspect.

In addition, we have that most of the cases were in stage II, it is an early stage and surgery can provide good results when performed correctly. Also, we have to consider that dogs with serosal, urethral, or ureteral invasion were excluded. This can be considered a selection bias, however, due to our knowledge and experience with ECT, we anticipated serious complications if we proceeded with the treatment of those cases. ECT mainly eliminates tumoral cells, as they are replicating faster. If the whole thickness of the bladder wall is replaced with tumoral tissue, then no normal tissue is present in that precise point. When the response is achieved, and the tumoral cells are eliminated, a perforation in the bladder wall can occur, producing the leakage of urine to the peritoneum. For those reasons, we consider it is mandatory to refrain from treating with ECT cases where serosal invasion is present. Future studies are needed to contrast the results of ECT with other local therapies.

## Methods

### Ethical aspects

This prospective clinical study was performed in accordance with the relevant guidelines and regulations of the Brazilian National Council for the Control of Animal Experimentation (CONCEA) and was approved by the Ethics Committee in the Use of Animals of the São Paulo State University “Júlio de Mesquita Filho” (UNESP), School of Agricultural and Veterinarian Sciences, Jaboticabal, São Paulo, Brazil (protocol no. 3848/20). Written informed consent to perform the treatment was obtained from all the dogs’ owners. All experiments were performed in accordance with relevant guidelines and regulations, complying with the ARRIVE guidelines.

### Study design and case selection

Twenty-one dogs with spontaneous bladder UC were enrolled in this prospective, longitudinal study. The dogs were presented to the Vet Câncer Oncologia e Patologia Animal (São Paulo, Brazil) between March 2016 and March 2021. ECT was offered as an alternative therapy when the owners refused other standard treatments such as surgery or medical therapy. The recommendations made by Campana et al.^68^ for reporting clinical studies of ECT were followed.

Before selection, the dogs underwent physical examination, complete blood count, serum biochemical analysis (creatinine, BUN, alkaline phosphatase, and ALT), and electrocardiogram. Three-view thoracic radiography and abdominal ultrasonography were also performed for screening of metastasis and for evaluating the bladder tumor. Dogs were classified according to the TNM clinical staging system defined by the World Health Organization for canine bladder tumors^2^.

Cases eligible for inclusion in the study had to meet the following criteria: (1) histopathological diagnosis of bladder UC; (2) T1 or T2 stage (without tumoral invasion of the bladder’s serosa layer); (3) absence of urethra and/or ureter involvement; (4) absence of regional and distant metastasis; and (5) rejection of the owners of other treatment modalities, after a thorough explanation of them. It is important to highlight that the invasion of the serosa, the urethra and/or the ureter was an exclusion criteria for the study, as there is a high risk of perforation after ECT in these cases.

### Surgical procedure

All procedures were performed under general anesthesia. For induction, propofol (Lipuro® 10 mg/ml; Braun, Melsungen, Germany) was administered intravenously at a dose of 5 mg/kg, followed by endotracheal intubation. Isoflurane (Genérico, Biochimico Laboratory, Itatiaia, Rio de Janeiro, Brazil) was used to maintain the anesthesia.

Laparotomy was performed using the ventral midline approach, followed by examination of the regional iliac lymph nodes, which were aspirated for intraoperative cytological evaluation (even if macroscopically normal) for staging purposes. Subsequently, the bladder was carefully isolated from the abdominal cavity, and cystotomy was performed. Following tumor visualization (Fig. 2A), incisional biopsies were performed and an intraoperative histopathological evaluation was performed.

During cystotomy, to minimize tumor cell spread and subsequent implantation in neighboring organs, the following criteria were met: (1) careful manipulation of the bladder; (2) exchange of surgical instruments and gloves after contact with the tumor; and (3) in the case of bladder eversion, contact of the inner bladder wall with any other intra-abdominal structure was avoided.

### Intraoperative histopathological evaluation

Intraoperative histopathological evaluation was performed using frozen sections. This technique comprised the steps of macroscopic analysis of the samples, cleavage, freezing with compressed CO_2_ and obtaining sections approximately 5 µm in thickness in a portable freezing microtome (Leica). The sections were then stained with toluidine blue and analyzed under a light microscope (Nikon YS2).

Intraoperative evaluation allowed the assessment of regional lymph node involvement, tumor diagnosis, degree of tumor infiltration on the bladder wall, and consequently, the definitive inclusion of the dogs (Fig. 1).

The tumors were classified according to the growth pattern (papillary vs. non-papillary), differentiation (conventional UC vs. divergent UC) and grade (low vs. high grade)^69,70^. Presence of inflammation and lymphatic invasion were also evaluated.

### Electrochemotherapy protocol

ECT was initiated by intravenous injection of BLM (Bleovet®) at a dose of 15,000 UI/m^2^, after dilution in 5 mL of saline solution. Eight minutes later, electrical pulses were applied over the tumor and the entire inner bladder wall (Fig. 2B). Pulses were triggered every time the bladder was punctured with the electrodes, to avoid cancer cell implantation at other bladder sites. The pulses were applied within a period of 40 min after drug injection, as recommended by Tellado, Mir and Maglietti^33^ in the Veterinary Guidelines for Electrochemotherapy of Superficial Tumors. ECT application is demonstrated in Supplementary Video 1.

Each train of pulses consisted of eight square bipolar pulses, 1000 V/cm, 100 μs long, at 5 kHz frequency, delivered by VETCP 125® (IMPLASTIC, São Paulo, Brazil). The pulses were delivered using a 6-needle electrode, which consisted of two sequences of three needles, with 5 mm distance between contralateral needles and a 3 mm distance between ipsilateral needles.

To monitor for immediate adverse effects, urinary output, and postsurgical pain, the dogs were hospitalized with a urethral catheter for at least 48 h. Anti-inflammatory drugs and analgesics used during hospitalization included tramadol hydrochloride (4 mg/kg/BID) and meloxicam (0,1 mg/kg/SID), both subcutaneously. Dogs were discharged with domiciliary prescription of tramadol hydrochloride (4 mg/kg/orally/BID) and piroxicam (0.3 mg/kg/orally/SID) for 3 more days.

### Case follow-up

Initial follow-up was performed 7, 14, 21, and 30 days after surgery. The treatment period was individualized for each case and was determined as the period between the day of the first ECT and 30 days after the last ECT session. If no further sessions were performed, regular follow-up was scheduled every 60 days for the first 6 months and every 90 days until a year after the treatment, which is considered the observation period. Physical examination, complete blood count, biochemical analysis, and abdominal ultrasonography were performed in each visit. Chest radiographs were obtained every 3 months to assess the presence of lung metastasis.

### Safety evaluation

The development of adverse events was assessed during ECT treatment, immediately after ECT treatment, during post surgical hospitalization, and at each follow-up visit. These events were graded according to the VCOG-CTCAE v2^49^ toxicity scale as follows: (1) mild, (2) moderate, (3) severe or medically significant, but not immediately life-threatening, (4) life-threatening consequences, or (5) death related to adverse events.

To determine the cause of death, necropsy was requested for all the cases. If the owner refused it, the cause of death was presumed according to the dogs’ clinical condition and blood and imaging tests.

### Quality of life

Quality of life was assessed before the first ECT procedure and at each visit using a questionnaire inspired by the Canine Owner-Reported Quality of Life, developed by Giuffrida et al.^71^.

The questionnaire contained 17 items related to observable behaviors commonly perceived by owners and was organized into four proposed factors: vitality, companionship, pain, and mobility. Each item was arranged as a sentence that described a common behavior, followed by a numerical analog scale (0–7). The owner was asked to circle the number of days in the previous week the behavior occurred.

Additional information obtained by the oncologist and owner at each follow-up visit was documented in the dog’s medical records.

### Response evaluation

According to the RECIST criteria^72^, the local response was assessed 30 days after each ECT session and the final local response was determined at the end of the treatment period, when no more ECT sessions were needed or the owner rejected them.

The local response and the need for additional ECT sessions were determined by ultrasound assessment performed at each visit by the same radiologist (Figs. 4 and 5). The owners were instructed to offer water ad libitum and not allow their animals to urinate for one hour before ultrasound examination. During the examinations, the dogs were positioned in dorsal recumbency with full urinary bladder repletion, according to the recommendations of Fulkerson and Knapp^73^ for bladder tumor imaging assessment.

New ECT sessions were performed when macroscopic evidence of the tumor was identified during ultrasound evaluation at least 30 days after the previous session. If a complete remission was identified, consent for an additional surgical procedure was requested from the owners, in order to confirm the absence of residual microscopic disease. If neoplastic foci were identified on intraoperative evaluation, a new ECT treatment was indicated.

Survival time was defined as the time between the first ECT and death, or the end of the study period. DFS was calculated only for cases who achieved CR. It was defined as the period between CR achievement and the day of local or distant relapse, death, or the end of the study. PFS was calculated only for dogs who achieved PR or stable disease, and was defined as the period from PR achievement (or from the first ECT, for stable disease cases) until progression or death. Finally, the DOR was calculated for all dogs who achieved CR, PR, or stable disease and was defined as the period between the final local response achievement until local or distant relapse/progression, death, or end of the study. Cases who obtained a PD after the ECT, were reported and redirected to other therapy. Dogs who developed progressive disease within the treatment period were excluded from the study and were directed to other treatment modalities.

### Statistical analysis

Statistical analyses were performed using MedCalc version 14.8.1. To analyze the influence of different factors on survival and disease-free survival, Kaplan–Meier curves were used, and significance was assessed using the Log-rank test. Fisher’s exact test was performed to determine the influence of different factors on the achievement of the response. Statistical significance was set at p < 0.01.

### Supplementary Information

Supplementary Video 1.

Supplementary Figure 1.

Supplementary Table 1.

Supplementary Legends.

## Data Availability

The data generated during this study are available from the corresponding author on reasonable request.
