# Supplementary material for: Evaluation of the safety and feasibility of electrochemotherapy with intravenous bleomycin as local treatment of bladder cancer in dogs
Source: Sci Rep. 2023 Nov 29;13:21078. doi: 10.1038/s41598-023-45433-4 (PMC10687251; doi:10.1038/s41598-023-45433-4)
Supplement: Supplementary file 2 — Supplementary Figure 1. [file 41598_2023_45433_MOESM2_ESM.docx]

**
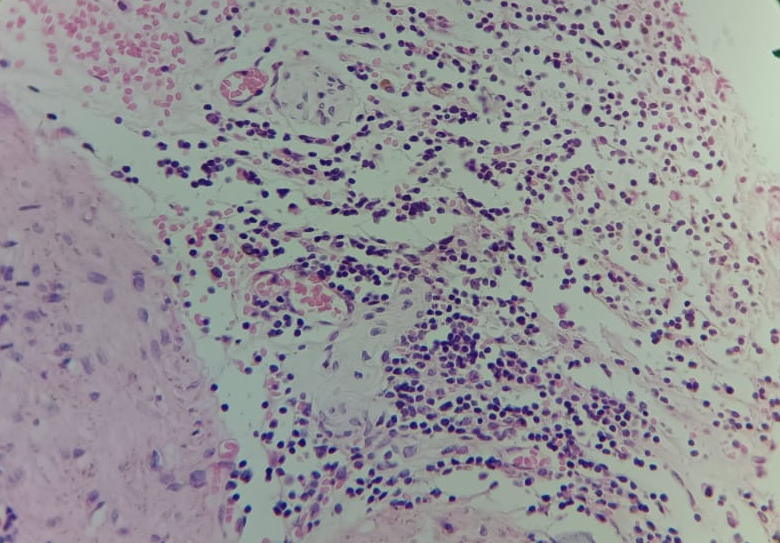
**

**Supplementary Figure 1*.*** Histological photomicrograph of the bladder muscular layer (case #17) at the time of ureteral stent placement (Hematoxylin and eosin, 40x magnification). Inflammatory lymphoplasmacytic infiltrate associated with histiocytes (black arrow), absence of tumor cells. Mucosa layer, affected by microscopic UC, was not represented in this sample.
