# Supplementary material for: Evaluation of the safety and feasibility of electrochemotherapy with intravenous bleomycin as local treatment of bladder cancer in dogs
Source: Sci Rep. 2023 Nov 29;13:21078. doi: 10.1038/s41598-023-45433-4 (PMC10687251; doi:10.1038/s41598-023-45433-4)
Supplement: Supplementary file 3 — Supplementary Table 1. [file 41598_2023_45433_MOESM3_ESM.docx]

**Supplementary Table 1.** TNM Clinical staging system for canine bladder cancer [2].

| T - Primary Tumor | | | | | |
| --- | --- | --- | --- | --- | --- |
| Tis | Carcinoma in situ |  |  |  |  |
| T0 | No evidence of a primary tumor | |  |  |  |
| T1 | Superficial papillary tumor | |  |  |  |
| T2 | Tumor invading the bladder wall, with induration | | | |  |
| T3 | Tumor invading neighboring organs (prostate, uterus, vagina ad pelvic canal) | | | | |
| N - Regional Lymph Node (Internal and External Iliac Lymph Node) | | | | | |
| N0 | No regional lymph node involvement | | |  |  |
| N1 | Regional lymph node involved | |  |  |  |
| N2 | Regional lymph node and juxtaregional lymph node involved | | | | |
| M - Distant Metastases | | | | | |
| M0 | No evidence of metastasis | |  |  |  |
| M1 | Distant metastasis present | |  |  |  |
