# Supplementary material for: Evaluation of the safety and feasibility of electrochemotherapy with intravenous bleomycin as local treatment of bladder cancer in dogs
Source: Sci Rep. 2023 Nov 29;13:21078. doi: 10.1038/s41598-023-45433-4 (PMC10687251; doi:10.1038/s41598-023-45433-4)
Supplement: Supplementary file 4 — Supplementary Legends. [file 41598_2023_45433_MOESM4_ESM.docx]

**Supplementary Video Legend**

**Video 1.** ECT treatment of a patient with bladder tumor. The ECT is applied over the tumor and the entire bladder inner wall. The manipulation should be very careful to avoid seeding tumoral cells in the abdominal wall.
